# Trends in reproductive, maternal, newborn and child health and nutrition indicators during five years of piloting and scaling-up of *Ananya* interventions in Bihar, India

**DOI:** 10.7189/jogh.10.021003

**Published:** 2020-12

**Authors:** Safa Abdalla, Yingjie Weng, Kala M Mehta1,, Tanmay Mahapatra, Sridhar Srikantiah, Hemant Shah, Victoria C Ward, Kevin T Pepper, Jason Bentley, Suzan L Carmichael, Andreea Creanga, Jess Wilhelm, Usha Kiran Tarigopula, Priya Nanda, Debarshi Bhattacharya, Yamini Atmavilas, Gary L Darmstadt, for the Ananya Study Group, Yamini Atmavilas, Bill and Melinda Gates Foundation, Delhi, India, Jason Bentley, Quantitaitve Sciences Unit, Department of Medicine, Stanford University, Stanford, CA, USA, Debarshi Bhattacharya, Bill and Melinda Gates Foundation, Delhi, India, Evan Borkum, Mathematica, Princeton, NJ, USA, Suzan L Carmichael, Department of Pediatrics, Stanford University School of Medicine, Stanford, CA, USA, Center for Population Health Sciences, Stanford University School of Medicine, Stanford, CA, USA, Indrajit Chaudhuri, Project Concern International, Patna, India, Andreea Creanga, Johns Hopkins Bloomberg School of Public Health, Baltimore, MD, USA, Gary L Darmstadt, Department of Pediatrics, Stanford University School of Medicine, Stanford, CA, USA, Center for Population Health Sciences, Stanford University School of Medicine, Stanford, CA, USA, Priyanka Dutt, BBM, Media, Action (India), New Delhi, India, Laili Irani, Population Council, New Delhi, India, CARE, India, Patna, India, Suneeta Krishnan, Bill and Melinda Gates Foundation, Delhi, India, Tanmay Mahapatra, CARE, India, Patna, India, Kala M Mehta, Center for Population Health Sciences, Stanford University School of Medicine, Stanford, CA, USA, Department of Epidemiology and Biostatistics, University of California San Francisco, San Francisco, CA, USA, Radhirani Mitra, BBM, Media, Action (India), New, Delhi, Wolfgang Munar, Department of Global Health, George Washington University Milken Institute School of Public Health, Washington, D.C., USA, Priya Nanda, Bill and Melinda Gates Foundation, Delhi, India, Kevin T Pepper, Department of Pediatrics, Stanford University School of Medicine, Stanford, CA, USA, Hina Raheel, Department of Pediatrics, Stanford University School of Medicine, Stanford, CA, USA, Anu Rangarajan, Mathematica, Princeton, NJ, USA, Niranjan Saggurti, Population Council, New, Delhi, India, Padmapriya Sastry, BBM, Media, Action (India), New, Delhi, India, Janine Schooley, Project Concern International, Patna, India, Hemant Shah, CARE, India, Patna, India, Sridhar Srikantiah, CARE, India, Patna, India, Usha Kiran Tarigopula, Bill and Melinda Gates Foundation, Delhi, India, Victoria C Ward, Department of Pediatrics, Stanford University School of Medicine, Stanford, CA, USA, Dilys Walker, Department of Obstetrics and Gynecology and Reproductive Services, Institute for Global Health Sciences, University of California San Francisco, San, Francisco, CA, USA, Yingjie Weng, Quantitative Sciences Unit, Department of Medicine, Stanford University, Stanford, CA, USA, Jess Wilhelm, Johns Hopkins Bloomberg School of Public Health, Baltimore, MD, USA

**Affiliations:** 1 Department of Pediatrics, Stanford University School of Medicine, Stanford, California, USA; 2 Quantitative Sciences Unit, Department of Medicine, Stanford University School of Medicine, Stanford, California, USA; 3 Department of Epidemiology and Biostatistics, University of California San Francisco, San Francisco, California, USA; 4 CARE India, Patna, India; 5 Center for Population Health Sciences, Stanford University School of Medicine, Palo Alto, California, USA; 6 Johns Hopkins Bloomberg School of Public Health, Baltimore, Maryland, USA; 7 Bill and Melinda Gates Foundation, Delhi, India

## Abstract

**Background:**

The *Ananya* program in Bihar implemented household and community-level interventions to improve reproductive, maternal, newborn and child health and nutrition (RMNCHN) in two phases: a first phase of intensive ancillary support to governmental implementation and innovation testing by non-government organisation (NGO) partners in eight focus districts (2012-2014), followed by a second phase of state-wide government-led implementation with techno-managerial assistance from NGOs (2014 onwards). This paper examines trends in RMNCHN indicators in the program’s implementation districts from 2012-2017.

**Methods:**

Eight consecutive rounds of cross-sectional Community-based Household Surveys conducted by CARE India in 2012-2017 provided comparable data on a large number of indicators of frontline worker (FLW) performance, mothers’ behaviours, and facility-based care and outreach service delivery across the continuum of maternal and child care. Logistic regression, considering the complex survey design and sample weights generated by that design, was used to estimate trends using survey rounds 2-5 for the first phase in the eight focus districts and rounds 6-9 for the second phase in all 38 districts statewide, as well as the overall change from round 2-9 in focus districts. To aid in contextualising the results, indicators were also compared amongst the formerly focus and the non-focus districts at the beginning of the second phase.

**Results:**

In the first phase, the levels of 34 out of 52 indicators increased significantly in the focus districts, including almost all indicators of FLW performance in antenatal and postnatal care, along with mother’s birth preparedness, some breastfeeding practices, and immunisations. Between the two phases, 33 of 52 indicators declined significantly. In the second phase, the formerly focus districts experienced a rise in the levels of 14 of 50 indicators and a decline in the levels of 14 other indicators. There was a rise in the levels of 22 out of 50 indicators in the non-focus districts in the second phase, with a decline in the levels of 13 other indicators.

**Conclusions:**

Improvements in indicators were conditional on implementation support to program activities at a level of intensity that was higher than what could be achieved at scale so far. Successes during the pilot phase of intensive support suggests that RMNCHN can be improved statewide in Bihar with sufficient investments in systems performance improvements.

**Study registration:**

ClinicalTrials.gov number NCT02726230.

Successful large-scale implementation of effective health interventions is a persistent challenge facing governments and non-governmental stakeholders in global health [1]. Major programs continue to be developed to overcome this challenge in places where progress in health outcomes is lagging [2,3]. One such program represents a partnership of the Bill and Melinda Gates Foundation (BMGF) with the Government of Bihar (GoB), India, formed in 2010 to accelerate the achievement of Bihar’s ambitious reproductive, maternal, newborn and child health and nutrition (RMNCHN) goals. The program is described in detail elsewhere [4,5]. Briefly, the program initially piloted a range of household, community, and facility-level interventions delivered across multiple government platforms through a number of initiatives to improve RMNCHN, aiming to identify the most effective interventions for scale-up. Those initiatives included the Integrated Family Health Initiative (IFHI) led by CARE (Cooperative for Assistance and Relief Everywhere) India to address issues of poor coverage and quality of services in family planning, skilled birth attendance, newborn care, prevention and management of neonatal sepsis, nutrition, and immunisation. An Incremental Learning Approach was implemented to improve frontline worker (FLW) effectiveness with intensified facilitation of training and supervision through externally placed facilitators. Quality improvement (QI) efforts employed a collaborative, team-based approach with mentored skill-building to improve the quality of RMNCHN services in public health facilities [6,7]. The Shaping Demand and Practices (SDP) initiative led by BBC Media Action used media campaigns and health messaging tools to increase demand for and adoption of priority health behaviours as well as to facilitate FLWs’ interactions with beneficiaries [8]. *Parivartan*, led by Project Concern International (PCI), advanced community mobilisation via self-help SHGs, integrating health modules into traditional SHGs to empower women to change behaviour and advocate for better quality of local RMNCHN services [9,10].

This first phase of the program relied on intensive support to governmental implementation of initiatives in eight “focus” districts by the aforementioned non-governmental organizations – CARE India, BBC Media Action and PCI – from 2011 to 2013. The intention of phased implementation was to identify those interventions that were successful, and to support the GoB to scale those across the rest of the state. The second phase, from 2014 to present, involved a shift from intensive NGO support to the provision of techno-managerial support through the Bihar Technical Support Program (BTSP), emphasising and facilitating government ownership and actions for implementation. The aim of the second phase, then, was to increase coverage and service quality at scale through health system strengthening and performance management by removing bottlenecks, providing planning and policy technical support, and building capacity via increased data utilisation and focus on outcomes [4]. This scale-up phase was characterised by significantly reduced external facilitation and implementation support, and relied on the government’s own capacity and actors to sustain the interventions and functions introduced and piloted in the previous phase.

The first phase was evaluated by two household surveys carried out by Mathematica, which are presented in detail elsewhere [5,11,12]. The baseline and “midline” surveys covered all 38 districts of Bihar, assessing changes in RMNCHN indicators in the eight focus districts compared to the 30 comparison districts where there was no program implementation. Concurrently, CARE India developed extensive surveys used primarily for the purposes of internal monitoring of key indicators during program implementation. These surveys were intended to provide robust district-level estimates for RMNCHN service coverage, quality, utilisation and beneficiary health behaviours at the household level, as well as to assess performance at sub-district level [13-15].

The phased implementation of the *Ananya* program offers a rich opportunity to study the impact of different intensities of interventions, scale, and implementation approaches, which can help to guide future efforts in introducing and scaling-up maternal and child health interventions in similar settings. We therefore aimed in this paper to examine how trends in RMNCHN indicators in the focus districts differed between the pilot and scale-up phases of the program, how they were impacted during the transition to scale-up, and how they compared between the initial eight focus districts and the other 30 districts of Bihar (where implementation started two years later) during scale-up so far.

## METHODS

### Design

We used data from the cross-sectional surveys collected by CARE India – referred to as the Community-based Household Surveys (CHS) – that spanned the full length of the program. There were nine survey rounds in 2012-2017. Round 1 was used as a pilot of the methodology and thus was not included in this analysis. However, because of this, program implementation had already commenced before the round 2 survey occurred, and thus, the first survey in the analysis may not represent a true baseline.

### Sampling

**Figure 1:**
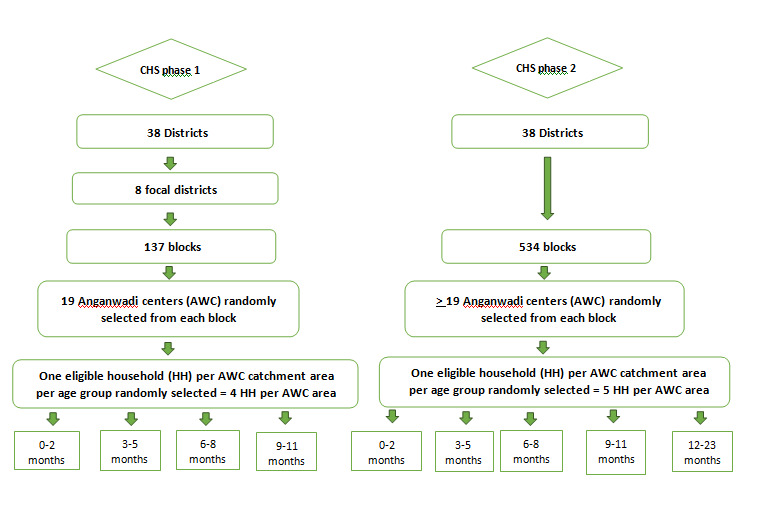
Sampling methodology of the Community-based Household Surveys (CHS), Bihar, 2012-2017.

Description of the survey design and sampling can be found in **Figure 1**. Rounds 2-5 were primarily designed to monitor the performance of sub-districts (blocks) in the eight focus districts during the first phase. Following a Lot Quality Assurance Sampling (LQAS) methodology, previously detailed elsewhere [16], a fixed number of 19 Anganwadi Centres (AWCs) (village-level facilities that provide basic education and nutrition services) were randomly selected from each of the 137 blocks constituting the eight focus districts. The AWC was the chosen unit because the catchment areas of the centres had limited variation in size, and a reliable sampling frame of AWCs was available. Using systematic random sampling to identify potentially eligible households, one mother of a child from each of the age groups of 0-2 months, 3-5 months, 6-8 months and 9-11 months was selected from each AWC’s catchment area, yielding about 2603 women per children’s age group per round. To minimise the recall period, the mothers of the children in each of the age groups responded to questions in different continuum of care domains that were the focus of interventions for that age group.

Rounds 6-9 were carried out during the second phase when program implementation had been scaled up to all 38 districts. A modified LQAS-like (LQAS+) methodology was used with a larger sample size aiming to obtain point estimates for each district with a precision of ±5%. These rounds also included mothers of children aged 12-23 months. The sample of mothers from each block was proportionate to the number of eligible women in the block, with a minimum of 19 women for each of the children’s age groups, yielding approximately 15 687 women per children’s age group per round.

### Data collection

An interview questionnaire was used to collect data on sociodemographic characteristics with specific modules for indicators relevant to each age group. Respondents completed an informed consent prior to the interview. Quality control was implemented from round 2 onwards with mandated spot checks and back-checks of 15% of the data by independent field supervisors. A metadata-base digital quality control system that was capable of tracking individual data collection devices in real time was established in 2016. Fieldwork in each survey round took about 2-3 months. Rounds 2-5 interviews were conducted by block coordinators, who were also facilitators of interventions, with a range of 4-6 months in between rounds. Rounds 6-9 were conducted with a range of 11-16 months in between rounds [4], and the data were collected by staff of Care India’s Concurrent Measuring and Learning unit which was functionally independent of implementation.

### Indicators

We assessed antenatal care (ANC), birth preparedness, delivery, postnatal care and breastfeeding indicators for children aged 0-2 months; and indicators of child nutrition/complementary feeding, child immunisation, and maternal use of contraception for children aged 9-11 months (Figure S1 in the **Online Supplementary Document**). We selected a total of 53 indicators from across the continuum of maternal and newborn care after a review by three independent members of the Stanford analytic team with expertise in maternal and child health and the conduct of field research, a thorough data quality check, and consultation with local partners. One indicator – use of a modern method of contraception – was not available in rounds 2-5, while three immunization indicators were not available in rounds 6-9. Although some elements in the continuum of care, such as ANC and exclusive breastfeeding, were not a particular focus of the program interventions, we still included them because they are key RMNCHN elements that may have been indirectly impacted. In addition to grouping indicators into continuum of care domains, we classified them within each continuum of care domain according to delivery platform: FLW performance, mother’s behaviour, and facility care or outreach service delivery (Table S1 in the **Online Supplementary Document**). Table S2 in the **Online Supplementary Document** describes the selected indicators.

### Data preparation

To enable comparison of levels of indicators and trends across the two phases of the program, we bridged the difference in sampling approaches between LQAS in CHS rounds 2-5 and LQAS+ in rounds 6-9. We linked the 137 blocks in the focus districts in rounds 2-5 with the districts and blocks in rounds 6-9 using the documented district and block names provided by CARE India. We then recreated sampling weights per block for each of rounds 2-9 and for each age group by dividing the number of eligible women by the number sampled. The number of eligible women per block was calculated using the National Family Health Survey (NFHS)-4 block-level census data [10] for the total population in the rural area multiplied by the district-level birth rate [11]. We used the linked block-IDs (137 in rounds 2-5 and 534 in rounds 6-9) as our sampling stratum and recalculated weights for each of the rounds as the study design sampling weights. Since nearly all primary sampling units (PSUs) – the AWCs –contributed only a single woman for each age group; there was no clustering at that level.

### Data analysis

Sociodemographic characteristics for the respondents were summarised with percentages for categorical variables and medians and inter-quartile ranges for continuous variables.

We made the following comparisons. First, we examined linear trends in the indicators: 1) in the first (pilot) phase between 2012 and 2014 using rounds 2-5 for districts (n = 8) that were the focus of the first phase, and 2) in the second (scale-up) phase between 2014 and 2017 using rounds 6-9, separately for the eight formerly focus districts and the 30 non-focus districts. Second, we compared indicator levels between round 5 and round 6 – the period of transition from the pilot phase to the scale-up phase – in the formerly focus districts. Third, we compared indicator levels at round 2 with the levels at round 9 in the formerly focus districts, to assess the net impact of the program over its full course to date. Fourth, we compared the indicator levels between the eight formerly focus districts and the 30 non-focus districts at round 6 – the beginning of the second phase – to contextualise subsequent trends in the second phase and factor any differences in the interpretation of those trends. For all of these comparisons, we used logistic regression models, taking into account the complex survey design and sample weights generated by that design, with the binary study indicators as the dependent variables. For analyses of linear trends, the independent variable was a time variable constructed by considering the start month of round 2 to be the baseline time with a value of 0. The time lapsed in years between baseline and the start month of each subsequent round was assigned to that round. The odds ratio for this time variable should therefore be interpreted as the relative change in odds of the indicator per year. For 2-round comparisons, round was a categorical variable. In an attempt to control for secular trends due to changing characteristics of the sample over time, models were adjusted for sociodemographic variables identified as potentially associated with RMNCHN: household size, combined religion-caste variable, mother’s age, mother’s literacy, number of children, gender of focal child, nuclear family, type of house, husband’s education, mother’s education and total household assets. We calculated an asset index as the total number of assets that the household had from among the list of assets asked in each round (Table S3 in the **Online Supplementary Document**).

Due to the large number of comparisons, we applied the False Discovery Rate (FDR) controlling procedure by Benjamini and Hochberg [17] using SAS (proc multtest) to all trend estimates together from all models, applying an upward adjustment to the *P* values. Family-wise type I error, alpha, was controlled at 0.05. Similar adjustment was carried out for the comparison of formerly focus districts and non-focus districts at the beginning of the second phase and the comparison between round 2 and round 9. Because adjustment of the *P*-values did not affect the conclusions about the trend for the vast majority of indicators, we report the results with the original confidence intervals, noting the few instances where FDR adjustment affected the result. All analyses were carried out in SAS 9.4 and accounted for complex survey design and sampling weights.

### Ethical considerations

Permission for access and terms of CHS data use were agreed with CARE India through a data sharing agreement and approved by the Stanford University Institutional Review Board (protocol number 39719). This study is part of the *Ananya* program which was registered with ClinicalTrials.gov number NCT02726230.

## RESULTS

### Sociodemographic characteristics of the surveyed population

**Table 1** depicts the sociodemographic characteristics of mothers of children aged 0-2 months (see also Table S4 in the **Online Supplementary Document**). In round 2, the median age of mothers of children aged 0-2 months was 24.1 (interquartile range (IQR) = 21.1-27.3) years, with little variation in subsequent rounds. Two-thirds of mothers (66.7%) had no education. Median household size was 6.7 (IQR = 4.9-9.5) and 76.6% of children had three or fewer siblings. Houses most commonly were *kutcha* (wood, mud, straw and dry leaves) (46.0%) followed by semi-*pucca* (38.9%) where either the roof or the walls were made of *pucca* materials such as burnt bricks and stone. Most of the respondents were Hindu non-Scheduled Caste/Scheduled Tribe (60.1%). Patterns were similar in subsequent rounds except for declines in *kutcha* houses and increases in semi-*pucca* housing, mothers with secondary education and median number of assets. Respondents in focus districts and non-focus districts were similar except for higher proportions in the latter of Hindu Scheduled Castes, *kutcha* housing, and men and women with secondary education. Similar patterns were observed among mothers of children aged 9-11 months (Table 2 and Table S5 in the **Online Supplementary Document**).

**Table 1:** Distribution of mothers of children aged 0-2 months by sociodemographic characteristics in 8 focus districts and 30 non-focus districts in rounds 2, 5, 6, and 9 of the Community-based Household Surveys in Bihar, India, 2012-2017 (frequency, weighted %)

Variable	Round 2	Round 5	Round 6		Round 9	
**Focus**	**Focus**	**Focus**	**Non-focus**	**Focus**	**Non-focus**
**Religion – caste, %:**						
Hindu, non-SC/ST*	1583 (60.1)	1587 (59.6)	2192 (62.5)	6882 (54.7)	2142 (61.4)	7081 (56.5)
Hindu, SC/ST	705 (26.4)	696 (27.1)	792 (23.6)	3399 (27.5)	876 (25.4)	3423 (27.6)
Non-Hindu	315 (13.5)	320 (13.2)	457 (14)	1965 (17.8)	423 (13.1)	1737 (15.9)
Missing						5 (0.03)
**Literate, %**	862 (33.3)	933 (35.4)	1296 (37.9)	4850 (39.8)	1617 (46.7)	5880 (48.3)
**Gender of focal child, %:**						
Female	1237 (47)	1264 (48.6)	1669 (48.5)	5878 (47.9)	1618 (47)	5889 (48)
Male	1366 (53)	1339 (51.4)	1772 (51.5)	6368 (52.1)	1823 (53)	6357 (52)
**Nuclear family, %**	890 (33.9)	889 (33.7)	1446 (42)	4980 (41.3)	1181 (34.7)	4113 (34.6)
**House type, %:**						
*Kutcha*	1159 (46)	1090 (43)	1164 (33.6)	4666 (39.1)	758 (22.1)	3471 (30.4)
Semi-*pucca*	1033 (38.9)	1077 (40.7)	1578 (47.1)	4656 (38.9)	2216 (65)	6960 (55.6)
Pucca	411 (15.1)	436 (16.3)	699 (19.3)	2924 (22)	467 (12.9)	1815 (14)
**Husband's education, %:**						
No education	1175 (44.6)	1113 (42.2)	1484 (42.9)	5254 (42.9)	1339 (38.2)	4920 (40)
Primary education (0-8 years)	756 (29.6)	797 (32)	960 (29.1)	2860 (24.6)	814 (24.8)	2421 (20.5)
Secondary education (9-12 years)	533 (20.3)	544 (20.1)	755 (20.6)	3194 (25.1)	838 (23.4)	3234 (25.6)
Higher education (>12 years)	106 (4.3)	114 (4.1)	196 (5.8)	812 (6.3)	227 (6.4)	1025 (8.3)
Missing	33 (1.4)	35 (1.5)	46 (1.6)	126 (1.1)	223 (7.1)	646 (5.6)
**Women's education, %:**						
No education	1741 (66.7)	1670 (64.6)	2152 (62.3)	7436 (60.5)	1832 (53.6)	6438 (52.4)
Primary education (0-8 years)	523 (20.2)	561 (21.7)	668 (20.1)	2167 (18.5)	696 (20.5)	2283 (19.1)
Secondary education (9-12 years)	298 (11.5)	308 (11.3)	511 (14.5)	2218 (17.6)	766 (21.9)	2910 (23.6)
Higher education (>12 years)	41 (1.6)	64 (2.4)	110 (3.2)	425 (3.4)	147 (4.1)	615 (4.9)
Missing						
**Number of children, %:**						
1	720 (27.6)	671 (26)	869 (24.5)	3268 (26.7)	934 (26.8)	3373 (27.8)
2	733 (28.4)	684 (26.6)	882 (25.3)	3256 (26.7)	958 (27.7)	3365 (27.7)
3	541 (20.6)	589 (22)	704 (20.8)	2469 (20.2)	703 (20.7)	2645 (21.4)
4+	609 (23.3)	659 (25.4)	986 (29.3)	3253 (26.4)	846 (24.8)	2863 (23.1)
Women’s age, years, median (IQR)*	24.1 (21.1-27.3)	24.1 (21.2-27.1)	24 (21.1-27)	24 (21.2-26.6)	22.4 (19.9-25.2)	22.7 (20.1-25.2)
**Number of assets, median (IQR)**	4.4 (2.7-6.6)	5.1 (3.4-5.1)	5.4 (3.6-7.5)	5.2 (3.4-7.7)	7.5 (5.5-9.9)	8.0 (5.7-9.4)
**Household size, median (IQR)**	6.7 (4.9-9.5)	6.7 (4.9-9.2)	6.4 (4.6-8.7)	6.2 (4.5-8.6)	6.5 (4.8-8.9)	6.5 (4.7-8.9)
**Number of children, median (IQR)**	1.8 (1-2.9)	1.9 (1-3)	2 (1-3.3)	1.9 (1-3.1)	1.8 (1-3)	1.8 (1-2.9)

IQR – interquartile range, SC – scheduled caste, ST – scheduled tribe

**Table 2:** Distribution of mothers of children aged 9-11 months by sociodemographic characteristics in 8 focus districts and 30 non-focus districts in rounds 2, 5, 6, and 9 of the Community-based Household Surveys in Bihar, India, 2012-2017 (frequency, weighted %)

Variable	Round 2	Round 5	Round 6		Round 9	
**Focus**	**Focus**	**Focus**	**Non-focus**	**Focus**	**Non-focus**
**Religion – caste, %:**						
Hindu, non-SC/ST*	1587 (60.1)	1533 (57.7)	2209 (62.9)	6964 (55.7)	2179 (62.7)	7167 (57.5)
Hindu, SC/ST	705 (27)	717 (27.7)	748 (22.4)	3387 (27)	858 (25.2)	3324 (26.5)
Non-Hindu	311 (12.9)	353 (14.6)	483 (14.6)	1896 (17.3)	404 (12.2)	1749 (16)
Missing						6 (0.03)
**Literate, %**	815 (31.1)	982 (37.6)	1286 (37.5)	4608 (38.4)	1528 (44.6)	5785 (47)
**Gender of focal child, %:**						
Female	1270 (49)	1243 (47.4)	1607 (46.9)	5748 (47)	1643 (47.9)	5794 (46.9)
Male	1333 (51)	1360 (52.6)	1833 (53.1)	6499 (53)	1798 (52.1)	6452 (53.1)
**Nuclear family, %**	1025 (38.9)	1015 (38.9)	1593 (45.5)	5740 (47.3)	1423 (41.6)	4896 (41.3)
**House type, %:**						
*Kutcha*	1087 (43.3)	1020 (40.4)	1210 (34.9)	4657 (39.2)	743 (22.1)	3288 (28.5)
Semi-*pucca*	1124 (42.3)	1100 (41.9)	1541 (46.5)	4612 (38.3)	2234 (65)	6967 (55.6)
*Pucca*	392 (14.4)	483 (17.7)	689 (18.6)	2978 (22.6)	464 (12.9)	1991 (15.8)
**Husband's education, %:**						
No education	1164 (45.2)	1092 (41.5)	1483 (42.4)	5314 (43)	1382 (39.7)	4812 (39.6)
Primary education (0-8 years)	738 (28.8)	746 (29.8)	981 (29.7)	2861 (24.6)	806 (24)	2446 (20.8)
Secondary education (9-12 years)	541 (20.2)	602 (22.5)	761 (21.3)	3171 (25.2)	779 (22)	3299 (26)
Higher education (>12 years)	106 (3.6)	135 (5)	169 (5.0)	768 (6.1)	255 (7.6)	1006 (7.9)
Missing	54 (2.2)	28 (1.2)	46 (1.6)	133 (1.1)	219 (6.7)	683 (5.7)
**Women's education, %:**						
No education	1788 (68.9)	1621 (62.4)	2161 (62.7)	7675 (61.9)	1927 (55.8)	6542 (53.9)
Primary education (0-8 years)	489 (19)	560 (21.6)	679 (20.4)	2228 (19.2)	683 (20.8)	2210 (18.6)
Secondary education (9-12 years)	279 (10.2)	341 (12.9)	516 (14.5)	1959 (15.8)	679 (19.2)	2844 (22.4)
Higher education (>12 years)	47 (1.8)	81 (3.1)	84 (2.4)	385 (3.1)	152 (4.2)	650 (5.1)
**Number of children, %:**						
1	724 (28)	772 (29.7)	923 (26.4)	3278 (27)	966 (27.8)	3418 (28.1)
2	694 (26.7)	673 (26.3)	902 (25.9)	3317 (27.2)	951 (28)	3438 (28.2)
3	520 (20)	551 (20.9)	687 (19.7)	2417 (19.6)	705 (20.5)	2616 (21.1)
4+	665 (25.3)	607 (23.1)	928 (27.9)	3235 (26.2)	819 (23.7)	2774 (22.6)
**Women's age, years, median (IQR)**	24.6 (21.6-27.9)	24.3 (21.5-27.4)	24.2 (21.6-27.1)	24.3 (21.6-27.1)	23.1 (20.3-25.6)	23.3 (20.6-25.9)
**Number of assets, median (IQR)**	4.1 (2.4-6.4)	5.2 (3.5- 7.5)	5.4 (3.5-7.6)	5.1 (3.3-7.6)	7.5 (5.4-9.9)	7.8 (5.6-10.5)
**Household size, median (IQR)**	6.3 (4.5-8.6)	6.3 (4.5-8.6)	5.9 (4.3-8.1)	5.8 (4.2-8.1)	5.9 (4.3-8.2)	5.9 (4.3-8.3)
**Number of children, median (IQR)**	1.8 (1-3)	1.8 (1-2.9)	1.9 (1-3.2)	1.8 (1-3.1)	1.8 (1-2.9)	1.8 (1-2.9)

IQR – interquartile range, SC – scheduled caste, ST – scheduled tribe

### Trends in RMNCHN indicators

**Figure 2:**
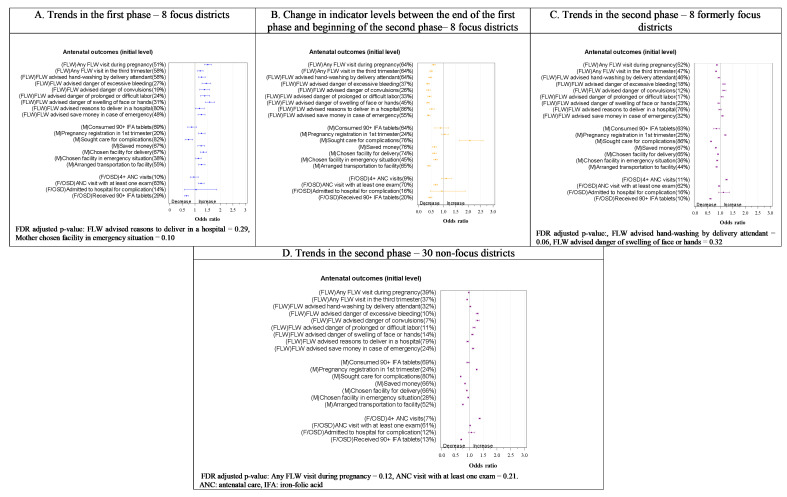
Trends in antenatal care and birth preparedness indicators in the first, transition and second phases of the Bihar program by delivery platform in focus and non-focus districts in Bihar, India, Community-based Household Surveys, 2012-2017. **Panel A**. Trends in the first phase – 8 focus districts. **Panel B**. Change in indicator levels between the end of the first phase and beginning of the second phase – 8 focus districts. **Panel C**. Trends in the second phase – 8 formerly focus districts. **Panel D**. Trends in the second phase – 30 non-focus districts. ANC – antenatal care, FDR – false discovery date, FLW – frontline worker, F/OSD – facility care and outreach service delivery, IFA – iron and folic acid, M – mother’s behaviour. Percentages in the y-axis labels are adjusted initial levels for each indicator in each phase. Odds ratios per year are presented on the x-axis. Indicator definitions can be found in Table S2 in the **Online Supplementary Document**.

**Figure 3:**
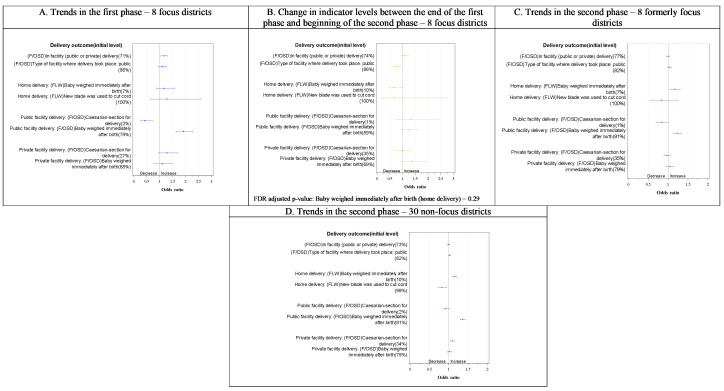
Trends in delivery (childbirth) care indicators in the first, transition and second phases of the Bihar program by facility vs home births and by delivery platform in focus and non-focus districts in Bihar, India, Community-based Household Surveys, 2012-2017. **Panel A**. Trends in the first phase – 8 focus districts. **Panel B**. Change in indicator levels between the end of the first phase and beginning of the second phase – 8 focus districts. **Panel C**. Trends in the second phase – 8 formerly focus districts. **Panel D**. Trends in the second phase – 30 non-focus districts. FDR – false discovery rate, FLW – frontline worker, F/OSD – facility care and outreach service delivery, M – mother’s behaviour,). Percentages in the y-axis labels are adjusted initial levels for each indicator in each phase. Odds ratios per year are presented on the x-axis. Indicator definitions can be found in Table S2 in the **Online Supplementary Document**.

**Figure 4:**
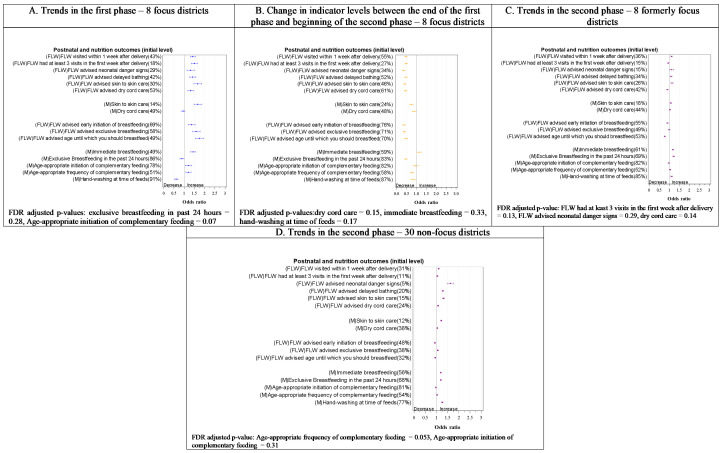
Trends in postnatal care and nutrition indicators in the first, transition and second phases of the Bihar program by delivery platform in focus and non-focus districts in Bihar, India, Community-based Household Surveys, 2012-2017. **Panel A**. Trends in the first phase – 8 focus districts. **Panel B**. Change in indicator levels between the end of the first phase and beginning of the second phase – 8 focus districts. **Panel C**. Trends in the second phase – 8 formerly focus districts. **Panel D**. Trends in the second phase – 30 non-focus districts. FDR – false discovery rate, FLW – frontline worker, F/OSD – facility care and outreach service delivery, M – mother’s behaviour. Percentages in the y-axis labels are adjusted initial levels for each indicator in each phase. Odds ratios per year are presented on the x-axis. Indicator definitions can be found in Table S2 in the **Online Supplementary Document**.

**Figure 5:**
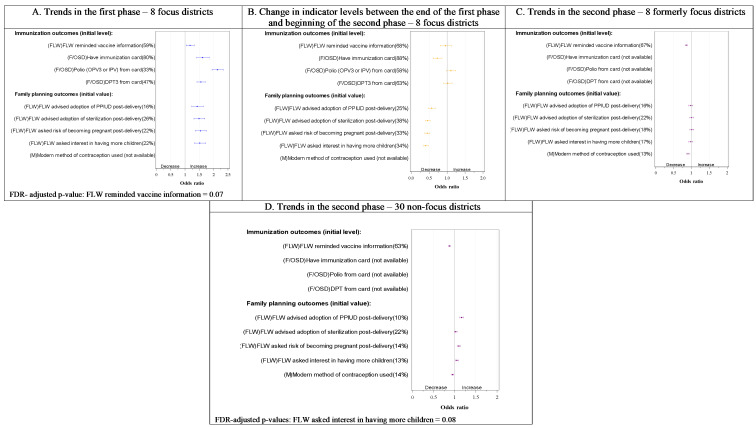
Trends in immunisation and family planning in the first, transition and second phases of the Bihar program by delivery platform in focus and non-focus districts in Bihar, India, Community-based Household Surveys, 2012-2017. **Panel A**. Trends in the first phase – 8 focus districts. **Panel B**. Change in indicator levels between the end of the first phase and beginning of the second phase – 8 focus districts. **Panel C**. Trends in the second phase – 8 formerly focus districts. **Panel D**. Trends in the second phase – 30 non-focus districts. FDR – false discovery rate, FLW – frontline worker, F/OSD – facility care and outreach service delivery, IPV – inactivated polio vaccine, M – mother’s behaviour, OPV3 – oral polio vaccine, 3 doses, PPIUD – post-partum Intrauterine Device. Percentages in the y-axis labels are adjusted initial levels for each indicator in each phase. Odds ratios per year are presented on the x-axis. Indicator definitions can be found in Table S2 in the **Online Supplementary Document**.

Overall, we observed a rising trend in 34 out of 52 indicators in the first phase in the focus districts (**Figure 2****,** **Figure 3****,** **Figure 4** and **Figure 5**, all Panel A). Subsequently, a relative decline in 33 out of 52 indicators took place between rounds 5 and 6 (transition from the first to the second phase) (**Figure 2**, **Figure 3**, **Figure 4** and **Figure 5**, all Panel B), including 28 of the 34 that improved in the first phase. Still, slightly more than half the indicators (28 out of 53 indicators) had significantly higher levels at the beginning of the first phase in the formerly focus districts than the non-focus districts. In the second phase, trends in the two groups of districts showed a mixed picture (**Figure 2****,** **Figure 3****,** **Figure 4** and **Figure 5****,** all Panels C and D), with an upward trend in some of the indicators (14/50 in formerly focus districts and 22/50 in the non-focus districts) and a downward trend in others (14/50 and 13/50 in formerly focus and non-focus districts, respectively). Overall, nine indicators had levels that were higher at round 9 than at round 2 in the formerly focus districts, 12 indicators were unchanged, while the remaining 28 indicators had lower levels at round 9 than at round 2.

#### Antenatal care and birth preparedness indicators

In round 2, 51% of women had received at least one FLW antenatal home visit during their last pregnancy. In the first phase (rounds 2-5), this level increased (odds ratio (OR) = 1.5, 95% confidence interval (CI) = 1.4-1.7) as did those of almost all other indicators of FLW performance (**Figure 2**, Panel A). Likewise, all indicators of antenatal mother’s behaviour related to birth preparedness improved (**Figure 2**, Panel A) except seeking care for complications, consumption of 90+ iron-folic acid (IFA) tablets, and choosing a facility in case of emergency. Following a statistically significant drop in most of these indicators in the transition phase (ie, between rounds 5 and 6), with the exception of a rise in seeking care for complications (**Figure 2**, Panel B), they remained unchanged or dropped further in the second phase (ie, rounds 6-9) although pregnancy registration and some indicators of FLWs’ advice on pregnancy complications improved (**Figure 2**, Panel C). At the beginning of the second phase, indicator levels were similar or significantly lower in the non-focus districts compared to the formerly focus districts, particularly those related to FLW performance (Figure S2 in the **Online Supplementary Document**), but more indicators of FLW performance increased in the non-focus districts (5 out of 9) than in formerly focus districts (3 out of 9) in the second phase (**Figure 2**, Panels C and D). Indicator levels in formerly focus districts exhibited either a decline or no net change in round 9 compared with round 2 (Figure S3 in the **Online Supplementary Document**). The only net rise was in the odds of women having at least four ANC visits (OR = 2.4, 95% CI = 2.0-2.9) and of women registering their pregnancy in the first trimester (OR = 2.5, 95% CI = 2.2-2.8).

#### Delivery

In round 2, around the beginning of the first phase, 71% of women gave birth in a health facility (**Figure 3**, Panel A). This increased by the end of the first phase to 74%, although with no significant change in the level of facility births that occurred in public facilities (**Figure 3**, Panels A). There was also an increase in weighing newborns immediately after birth in public facilities but not in private facilities. Caesarean births in public facilities were initially much less than in private facilities in round 2 and declined in the first phase, with no statistically significant change in those in private facilities. During the transition from the first to the second phase, the only significant decline was in deliveries taking place in public facilities (**Figure 3**, Panel B). At the beginning of the second phase (round 6), the levels of most childbirth indicators were similar in the non-focus districts to those in the formerly focus districts (Figure S2 in the **Online Supplementary Document**). In the second phase, weighing the baby immediately after birth increased in both home deliveries and public facility deliveries in formerly focus districts (**Figure 3**, Panel C) and in non-focus districts (**Figure 3**, Panel D). There was no evidence of a net difference in indicator levels between 2012 (round 2) and 2017 (round 9) in the focus districts (Figure S3 in the **Online Supplementary Document**) except for higher odds of facility deliveries and higher odds of weighing newborns immediately after home birth and public facility birth in round 9 compared with round 2 (OR = 2.0, 95% CI = 1.3-2.9 and 6.0, 95% CI = 4.5-8.0), and lower odds of public facility caesarean births.

#### Postnatal care

At round 2, 43% of women in the focus districts were visited at home by a FLW in the first postnatal week, and 18% received at least three visits within the first week. The first phase in the focus districts was marked by a rise in appropriate postnatal care including home visits and advice from FLWs. Skin-to-skin care showed a rising trend (OR = 1.6, 95% CI = 1.5-1.9) (**Figure 4**, Panel A). In the transition phase (**Figure 4**, Panel B), all these indicators declined. At the beginning of the second phase (round 6), indicator levels were all statistically significantly lower in the non-focus districts (Figure S2 in the **Online Supplementary Document**). In the second phase (**Figure 4**, Panel C), three of the six indicators of FLW performance (a visit in the first week after delivery, advising delayed bathing, and advising skin-to-skin care) in addition to skin-to-skin care practice showed statistically significant increases in the focus district, while the levels of all six indicators of FLW performance increased in the non-focus districts (**Figure 4**, Panel D). Only skin-to-skin care practice showed a net increase between rounds 2 and 9 in focus districts (Figure S3 in the **Online Supplementary Document**).

#### Nutrition

Exclusive breastfeeding of an infant aged 0-2 months in the 24 hours preceding the survey interview was reported by 86% of mothers, 78% of mothers of 9-11-month-olds initiated complementary feeding at the appropriate age of 6-8 months, and 51% gave complementary feeding at the age-appropriate frequency in the focus districts at round 2. During the first phase (**Figure 4**, Panel A), all three indicators of FLW performance – advice on early initiation of breastfeeding, exclusive breastfeeding, and age until which the mother should breastfeed – registered rising trends, along with two of the five indicators of feeding practices: immediate breastfeeding (OR = 1.4, 95% CI = 1.3-1.6), and age-appropriate frequency of complementary feeding (OR = 1.2, 95% CI = 1.1- 1.3). However, during this period, hand-washing between feeds declined and exclusive breastfeeding did not change. In the transition phase (**Figure 4**, Panel B), most indicators in this domain declined in the focus districts. During the second phase (**Figure 4**, Panel C), more indicators of feeding practices – immediate breastfeeding, exclusive breastfeeding, age-appropriate frequency of complementary feeding, and hand-washing between feeds – improved while those reflective of FLW performance remained unchanged or decreased. The non-focus districts started the second phase at similar or lower levels than the formerly focus districts (Figure S2 in the **Online Supplementary Document**) and followed similar trends. In the formerly focus districts, most indicators in this domain had lower levels in round 9 compared with round 2 except for immediate breastfeeding and age-appropriate frequency of complementary feeding (Figure S3 in the **Online Supplementary Document**).

#### Immunisation

Immunisation cards were available for 80% of children 9-11 months old in the focus districts at round 2. Less than half of the children had up-to-date trivalent diptheria-pertussis-tetanus (DPT) or polio immunisation that could be confirmed by an immunisation card (47% and 33% respectively). Having an immunisation card increased during the first phase (OR = 1.6, 95% CI = 1.4-1.8) as did up-to-date immunisations (**Figure 5**, Panel A). During transition to the second phase (**Figure 5**, Panel B), there were no statistically significant changes in any of the indicators except for a decline in having an immunisation card. In the second phase (**Figure 5**, Panel C), FLW reminding mothers of the next vaccination declined, ending up at a lower level compared with the beginning of the first phase (Figure S3 in the **Online Supplementary Document**) in the formerly focus districts. This indicator showed a similar decline in the non-focus districts (Figure S2 in the **Online Supplementary Document**).

#### Family planning

Only 16%-26% of postpartum mothers reported that a FLW advised them about various aspects of family planning at the beginning of the first phase in the focus districts. Those levels increased significantly in the first phase (**Figure 5**, Panel A), but declined significantly in the transition to the second phase (**Figure 5**, Panel B), with no evidence of a further change in the second phase (**Figure 5**, Panel C). The non-focus districts started the second phase at lower levels than the formerly focus districts with respect to three of these four FLW performance indicators: FLW asking interest in having more children, FLW advice about risk of becoming pregnant post-delivery, and FLW advising postpartum intrauterine device post-delivery for contraception (Figure S2 in the **Online Supplementary Document**). There was a significant increase in the second phase in the levels of the latter two. By round 9, most of those FLW performance indicators had lower levels than in round 2 in the focus districts (Figure S3 in the **Online Supplementary Document**). Use of a modern method of contraception (collected only in the second phase, rounds 6-9) had almost similar levels in focus and non-focus districts and declined in both groups in the second phase.

## DISCUSSION

In this analysis, we examined trends in RMNCHN indicators throughout the *Ananya* program from 2012-2017, observing what appeared to be a rise in many indicators in the pilot or first phase in the focus districts during intensive implementation. This was particularly prominent for indicators related to FLW performance with respect to visiting and advising mothers, in addition to mothers’ behaviours in birth preparedness that were specific areas of focus of the program. During the transition between the first and the second phase, most indicators declined as program activities declined in intensity in preparation for scale-up, again more remarkably in indicators of FLW performance. Despite these observations, most of these indicators remained at a higher level in the focus districts compared with the non-focus districts. However, during the second phase of state-wide implementation with lower-intensity TSU support, there was little change in the indicators in both the formerly focus and non-focus districts, although there were some improvements in indicators of FLW advice provision. In both formerly focus and non-focus districts, behaviours of mothers in the area of nutrition also improved but significant declines were evident in reported birth preparedness practices in the antenatal period.

Overall, the findings portray a picture of gains made mainly during intensive ancillary support by NGOs in the first phase, which were largely lost during the transition to scale-up, with a mix of some gains and some declines in the scale-up phase. Although comparison districts were lacking in the scale-up phase, the variation in trends at different intensity levels of the program, with the drop in most indicators during withdrawal of intensive support at transition, implies an association of those trends with the intensity of implementation of program activities. In particular, we note that among the three primary delivery platforms, FLW performance seemed to be the most sensitive to changes in the intensity of implementation, support and facilitation, likely because FLW performance indicators were the most proximal to the FLW-focused programmatic support [4]; their advising role in particular could potentially be less affected by other factors such as wider health system performance or beneficiaries’ demand for health care.

Although CARE India was extensively involved in the provision of techno-managerial support to the GoB during scale up starting in 2014, but not in direct health care delivery, the findings align with multiple other programs where the success of interventions that were heavily supported by NGOs was not robustly sustained by government systems while scaling up [18]. A commonly suggested reason is that NGOs have their own behavioural models and modes of technical supervision, training, leadership and staff management [18] which collectively constitute an organisational culture that cannot be easily replicated and sustained by governments across large populations. The trends elucidated by our analysis, particularly for FLW performance, may similarly reflect a sensitivity to the intensity of mentoring and supervision of health care workers or to the organisational culture, including drivers of motivation that are conveyed by an NGO compared to government systems. Studies of FLW performance in Bihar and Uttar Pradesh found that supervision was an important determinant of performance and motivation and that in those states, government supervisors of FLWs focused on their vertical accountability role, with limited supportive supervision and mentoring and little agency to enforce positive or negative sanctions [19,20]. Our qualitative research in non-focus districts in Bihar suggested that efforts to nurture and sustain ASHAs’ intrinsic motivation while addressing deficiencies in supportive supervision are necessary for improving the performance of Bihar’s ASHA program [19].

Health system strengthening, a feature of the second phase of the program, is critical for overcoming barriers to scale-up [1,21], but naturally requires a longer period of time to show results [22]. The GoB may require more time to deliver such a program at the required level of intensity and at scale as they address chronic weaknesses in major health system components, such as workforce motivation and performance, which could limit the capacity required for health impact at scale [19,23,24]. Specific events that took place in the second phase, such as staff reimbursement issues and industrial action might have also delayed progress in some areas. Another possible compounding factor was program complexity, with a range of heterogeneous interventions and delivery platforms despite the adaptations made to the interventions to suit the capacity of the health system [1]. As a result, three years of scale-up, especially at lower intensity, might not have been sufficient for the government to fully integrate the range of interventions at a large scale with the quality and saturation required for measureable improvements in outcomes. A lengthier phase prior to scale-up may also have permitted sufficient testing of the ability of the health system to absorb and sustainably deliver the interventions at scale and at a similar level of intensity as the first phase [21]. Raising the level of intensity of implementation close to that in the first phase, at least in parts of the state where it is needed the most, may achieve faster improvements but may have cost implications. Further analysis of the geospatial heterogeneity of system performance may aid in targeting resources and increases in intensity of implementation inputs to areas with the greatest need and potential for improvements.

It is recognised that periods of intense external support to program implementation carry the risk of unintentionally weakening health systems [25]. While the program was designed for identification of interventions for implementation system-wide, it should be noted that there were less intentional efforts in the first phase to strengthen the system across the full range of its components, apart from a separate cross-sectoral initiative that was being implemented simultaneously that was not specific for RMNCHN [26]. Although we do not have systematic data on the health system capacity over the duration of the program, the similarity in trends during scale-up between formerly focus districts and non-focus districts indicates that the system in those focus districts was at least not weakened by the initial intense external support. Neither, however, were these districts more responsive to the lower level inputs provided during the scale-up phase. In addition, indicators in areas that were not the focus of the program – such as having at least four ANC check-ups and practicing exclusive breastfeeding – did not appear to be particularly negatively impacted, even showing favourable trends in the second phase.

We have employed a rigorous approach in our data analysis, recalculating sampling weights across rounds to enable comparison between the two phases of the program as well as overall for the program between rounds 2 and 9. We also adjusted for the full complement of key sociodemographic variables in our analyses in order to minimise the effects of economic and social changes unrelated to the *Ananya* program (eg, rising maternal literacy, improving economic conditions). Given the secular improvements noted in a number of indicators in the first phase of implementation through the Mathematica difference-in-difference analysis [5], it is likely that these external factors were not fully accounted for in the present analysis using CHS. We lacked data from non-focus districts for comparison with focus districts in the first phase, as well as data from other states of India that could serve as controls during the second phase. The influence of state-wide factors external to *Ananya* such as changes to conditional cash transfer programs for facility deliveries and health worker strikes could not be quantified.

We note some limitations of our analysis. Round 2, which we treated in our analysis as the baseline, was carried out after some interventions had already begun to be implemented, and therefore the levels we designated as initial values may not have been representative of pre-intervention levels. The data relied on maternal self-report for the majority of indicators. Finally, this analysis was meant to be a high-level overview of changes in RMNCHN indicators during the program and more detailed explorations are needed to understand the successes and failures of the large and complex catalytic efforts encompassed by the program.

## CONCLUSION

The *Ananya* program has shown some promising results with the available public health workforce in Bihar, but those results seemed conditional on the intensity of implementation support. The required level of capacity did not appear to have been yet achieved by the public health system at scale over the period of time included in this analysis. Yet, the rising trends observed during the first phase of the program suggest that it is possible to improve RMNCHN in Bihar, if the right investments are made to sustain the type and intensity of support required across the various health system components.

## Additional material

Online Supplementary Document
