# Supplementary material for: Trends in reproductive, maternal, newborn and child health and nutrition indicators during five years of piloting and scaling-up of Ananya interventions in Bihar, India
Source: J Glob Health. 2020 Nov 30;10(2):021003. doi: 10.7189/jogh.10.021003 (PMC7757843; doi:10.7189/jogh.10.021003)
Supplement: Online Supplementary Document [file jogh-10-021003-s001.pdf]

Supplementary Figure 1: Community-based Household Survey samples used in the study

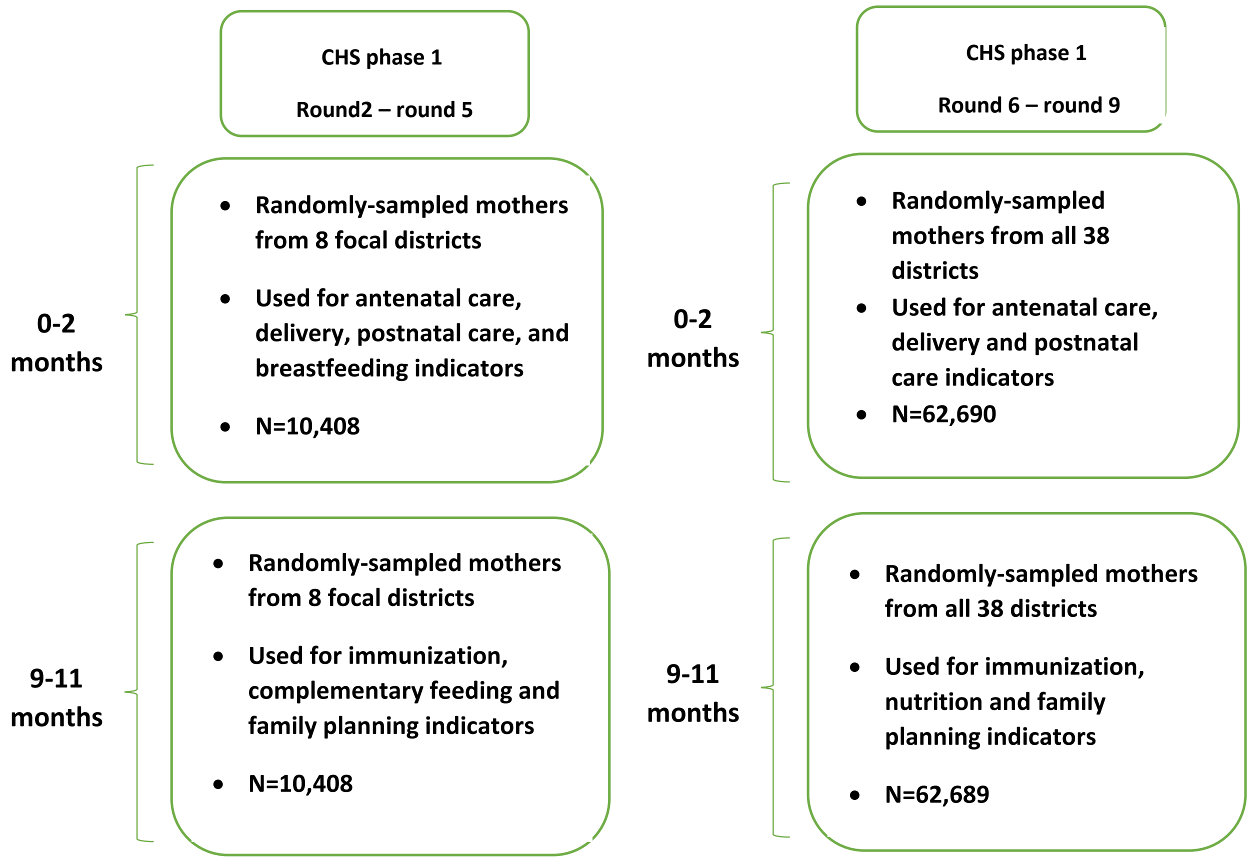

Supplementary figure 2: Comparison of RMNCHN indicators in the 30 non-focus districts to the formerly focus districts by continuum of care and delivery platform in round 6 of Community-based Household Surveys in Bihar, India (FLW: Frontline worker performance, M: Mother's behaviour, F/OSD: facility care and outreach service delivery).

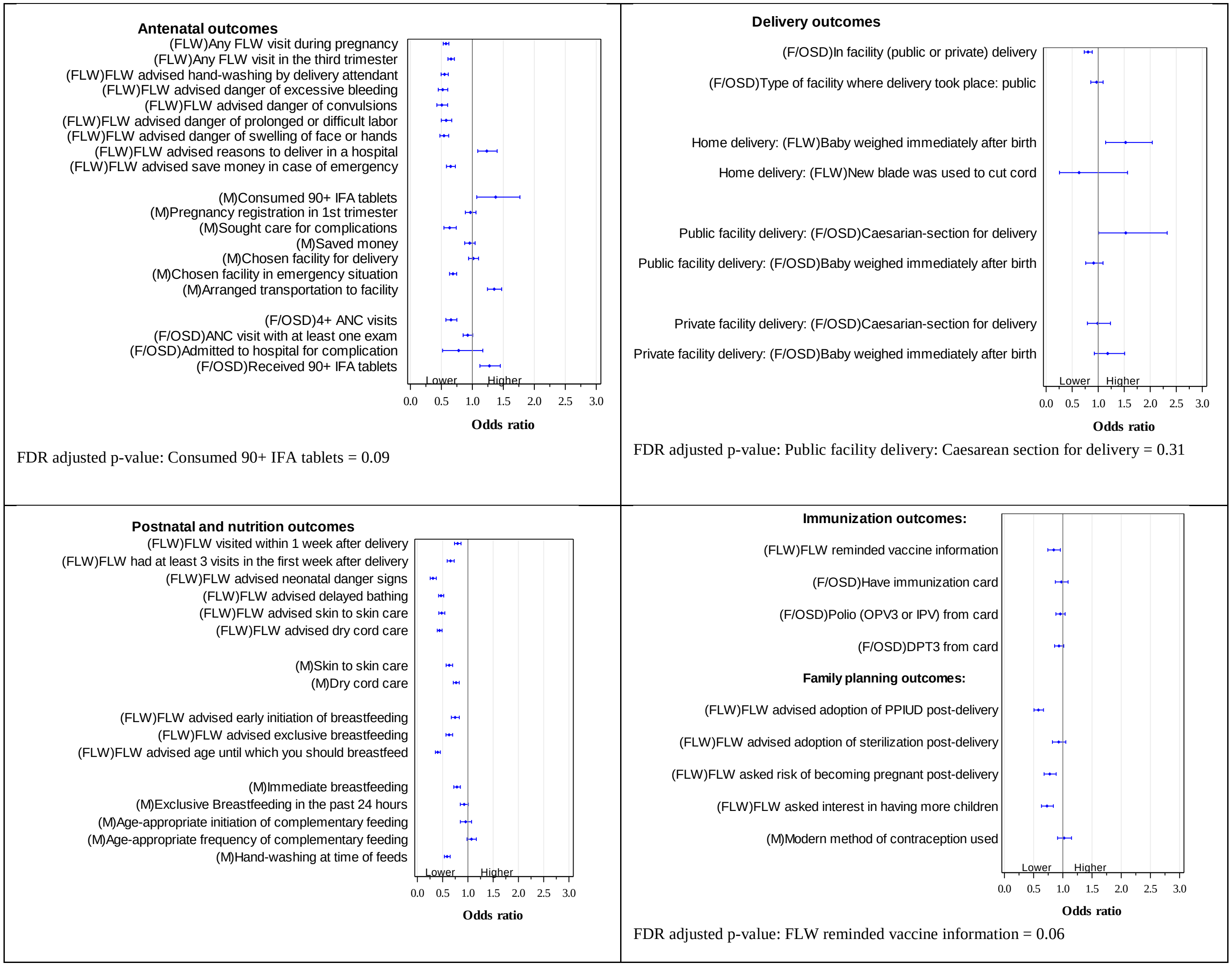

Supplementary Figure 3: Comparison of RMNCHN indicators in focus districts between rounds 2 and 9 of Community-based Household Surveys in Bihar, India. (FLW: Frontline worker performance, M: Mother's behaviour, F/OSD: facility care and outreach service delivery). Percentages in the y-axis labels are adjusted initial levels for each indicator in the focus districts.

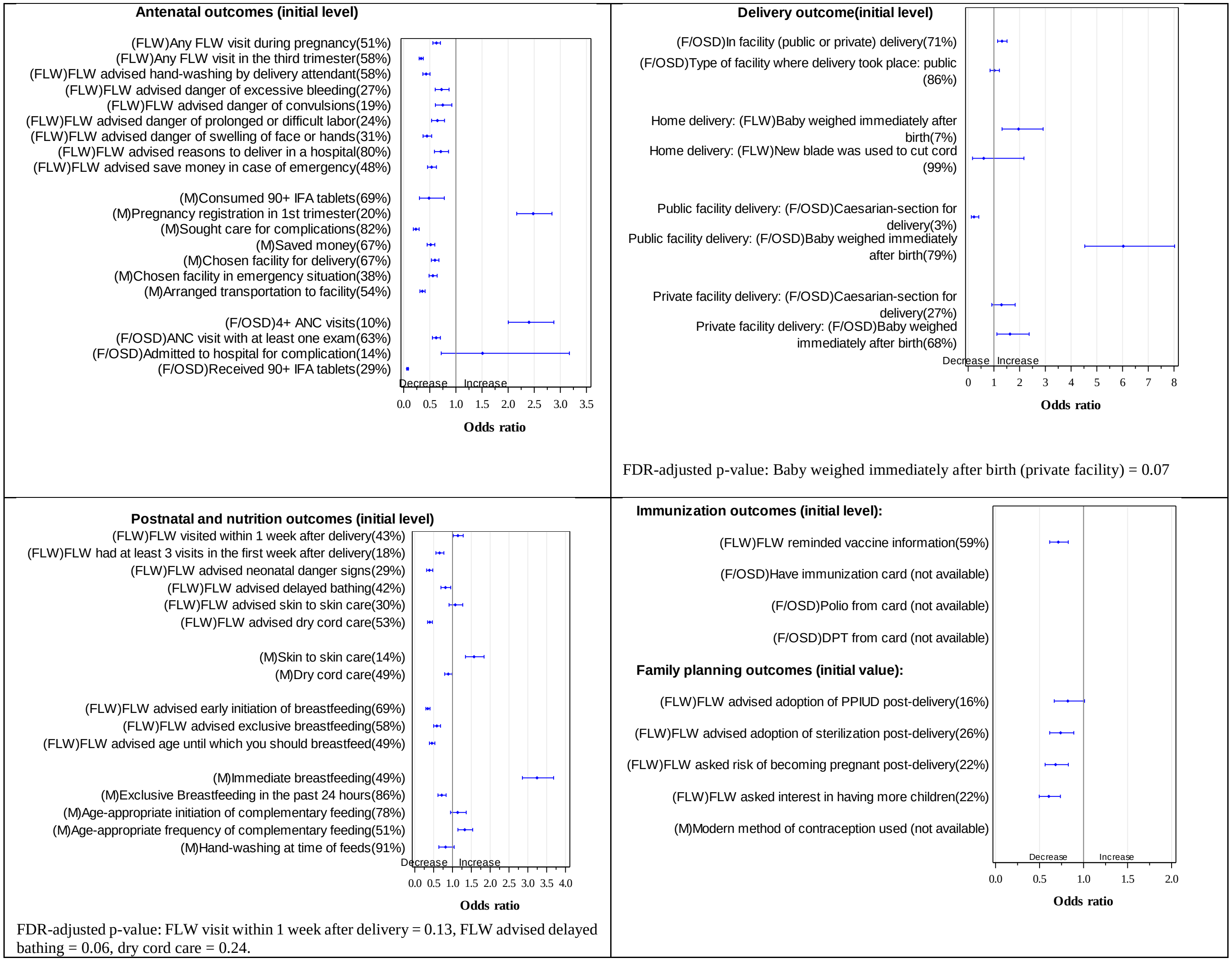

FDR-adjusted p-value: Baby weighed immediately after birth (private facility) = 0.07

FDR-adjusted p-value: FLW visit within 1 week after delivery = 0.13, FLW advised delayed bathing = 0.06, dry cord care = 0.24.

Supplementary Table 1: Categorisation of RMNCHN indicators by continuum of care domain and delivery platform

Continuum of care domain	Frontline Worker performance	Mother’s behaviour	Facility/outreach service delivery
**Antenatal care**	FLW visited any time during pregnancy for health counselling	Consumed 90+ IFA tablets	
	FLW visited any time in the third trimester	Pregnancy registration in the third trimester	ANC with at least one examination
		Sought care for complications	4+ ANC visits (see above)
	FLW advised hand-washing by delivery attendant	Saved money	Admitted to hospital for complication
	FLW advised danger of excessive bleeding	Chosen facility for delivery	Received 90+ IFA tablets
	FLW advised danger of convulsions	Chosen facility in emergency situation	
	FLW advised danger of prolonged or difficult labor	Arranged transportation to facility	
	FLW advised danger of swelling of face or hand		
	FLW advised save money in case of emergency		
	FLW advised reasons to deliver in a hospital		
**Delivery**			All deliveries:
			Gave birth in a facility
			Gave birth in a public facility
			Home delivery:
			Baby weighed immediately after birth<br>New blade was used to cut cord<br>Facility delivery (stratified by public and private facility):<br>Caesarean-section for delivery<br>Baby weighed immediately after birth
**Postnatal care**	FLW visited within 1 week after delivery	Skin to skin care	
	FLW had at least 3 visits in the first week after delivery	Dry cord care	
	FLW advised delayed bathing		
	FLW advised skin to skin care		
	FLW advised dry cord care		
	FLW advised neonatal danger signs		
**Complementary feeding/nutrition**	FLW advised early initiation of breastfeeding	Immediate breastfeeding	
	FLW advised exclusive breastfeeding	Exclusive Breastfeeding in the past 24 hours	

Continuum of care domain	Frontline Worker performance	Mother's behaviour	Facility/outreach service delivery
	FLW advised age until which you should breastfeed exclusively	Age-appropriate initiation of complementary feeding<br>Age appropriate frequency of complementary feeding<br>Hand-washing at time of feeds	
**Immunization**	FLW reminded vaccine information		Have immunization card<br>Polio (OPV3 or IPV) from card<br>DPT3 from card
**Family planning**	FLW asked interest in having more children<br>FLW asked risk of becoming pregnant post-delivery<br>FLW advised adoption of sterilization<br>FLW advised adoption of PPIUD post-delivery	Modern method of contraception used	

Supplementary table 2: RMNCHN indicators utilized in analyses of Community-based Household Surveys

Indicator	Child age group (months)	Indicator description
4+ ANC visits	0-2	Received four or more antenatal check-ups during last pregnancy
ANC with at least one exam	0-2	Had antenatal check-up where at least one of the following examinations was done at least once: blood pressure, abdominal examination, weight, height, blood examination, urine examination, breast examination and ultrasound examination.
Saved money	0-2	Mother put aside money specially for use during delivery or in an emergency
Chosen facility for delivery	0-2	Mother made plans to deliver in a facility in her last pregnancy.
Chosen facility in emergency situation	0-2	Mother planned to go to a different facility in the case of a serious emergency during pregnancy or delivery
Arranged transportation to facility	0-2	Mother identified in advance the vehicle she would use to reach health facility
Received 90+ IFA	0-2	In the last pregnancy, mother received at least 90 tablets of Iron and Folic Acid (IFA)
Consumed 90+ IFA tablets	0-2	In the last pregnancy, mother consumed at least 90 tablets if she received at least 90 tables of IFA.
Pregnancy registration in the third trimester	0-2	Mother registered her pregnancy no later than in the first trimester.
Sought care for complications	0-2	Mother consulted anybody or sought treatment if she had experienced excessive bleeding or convulsions
Admitted to hospital for complication	0-2	Mother admitted in the hospital/health center if she had experienced excessive bleeding or convulsions
FLW visited any time during last pregnancy	0-2	ASHA/AWW/ANM ever visited the mother to talk to her about her health or her baby's health
FLW visit during last tri-mester	0-2	ASHA/AWW/ANM ever visited the mother to talk to her about her health or her baby's health at least once in the last trimester of pregnancy
FLW advised hand-washing by delivery attendant	0-2	ASHA/AWW/ANM told the mother that the person who conducts her delivery should WASH her/his HAND before assisting you
FLW advised danger of excessive bleeding	0-2	ASHA/AWW/ANM told the mother about the danger of excessive bleeding during pregnancy, during delivery or after delivery
FLW advised danger of convulsions	0-2	ASHA/AWW/ANM told the mother about the danger of convulsions
FLW advised danger of prolonged or difficult labour	0-2	ASHA/AWW/ANM told the mother about the danger of prolonged or difficult labor
FLW advised danger of swelling of face or hand	0-2	ASHA/AWW/ANM told the mother about the danger of swelling of the face or hands
FLW advised reasons to deliver in a hospital	0-2	ASHA/AWW/ANM told the mother about the need to deliver in a hospital
FLW advised save money in case of emergency	0-2	ASHA/AWW/ANM told the mother about the need to keep some money aside for delivery or in case of emergency.

Indicator	Child age group (months)	Indicator description
FLW asked interest in having more children	0-2	ASHA/AWW/ANM enquired from the mother or her husband whether they intended to have more children
FLW asked risk of becoming pregnant post-delivery	0-2	ASHA/AWW/ANM told the mother about the risk of becoming pregnant soon after delivery if she did not use a method to avoid becoming pregnant
FLW advised advised adoption of sterilization	0-2	ASHA/AWW/ANM told the mother that she can adopt TL (female sterilization) immediately after delivery to avoid any further pregnancy
FLW advised adoption of PPIUD post-delivery	0-2	ASHA/AWW/ANM told the mother that she can adopt PPIUD immediately after delivery to delay next pregnancy
Place of delivery: In a facility (public or private)	0-2	Delivery took place in Medical college, District hospital, SDH, PHC, APHC/subcenter, or private hospital or clinic
Facility delivery took place in a public facility	0-2	Facility delivery took place in Medical college, District hospital, SDH, PHC, or APHC/subcenter
New blade was used to cut cord	0-2	New blade used to cut the cord
FLW visited day or next of delivery/Return from hospital	0-2	ASHA, ANM, AWW visited mother's home on the day of delivery (for home delivery) / on the first day after she returned home (for facility delivery)
FLW visited within 1 week after delivery	0-2	At least one visit by any FLW (ASHA, ANM, AWW) in the first week after delivery
FLW had at least 3 visits in the first week after delivery	0-2	At least three visits by any FLW (ASHA, ANM, AWW) in the first week after delivery
Baby weighed immediately after birth	0-2	Baby weighed in the hospital (for facility delivery) / baby first weighed after birth (home delivery)
FLW advised neonatal danger signs	0-2	ASHA/AWW/ANM told the mother that her child might have following signs/symptom, which would mean that her child had a serious illness?<br>a. May become unconscious or difficult to awaken<br>b. May develop difficulty in breathing or starting rapid breathing?<br>c. May loss interest in breastfeeding or may stop breastfeeding?<br>d. May become cold to touch?
FLW advised early initiation of breastfeeding	0-2	ASHA/AWW/ANM told the mother that the newborn should be breastfed as soon as possible after delivery
FLW advised exclusive breastfeeding	0-2	ASHA/AWW/ANM told the mother that the baby should not be given anything other than breastmilk up to the age of six months
FLW advised age until which you should breastfeed	0-2	ASHA/AWW/ANM told mother to breastfeed the baby until a particular age
FLW advised delayed bathing	0-2	ASHA/AWW/ANM told the mother that the baby should not be bathed for a few days (48 hours) after birth.
FLW advised skin to skin care	0-2	ASHA/AWW/ANM told the mother that the baby should be placed naked on her bare chest immediately after birth
FLW advised dry cord care	0-2	ASHA/AWW/ANM told the mother that nothing should be applied to the cord stump at birth

Indicator	Child age group (months)	Indicator description
FLW reminded vaccine information	0-2	ASHA/AWW/ANM told the mother on what date or month the child was due for his next vaccine
Immediate breastfeeding	0-2	Breastfeeding initiated within one hour after birth
Skin to skin care	0-2	Mother was advised by anyone at any time during or after delivery to keep the baby naked on her bare chest, next to her skin, and practiced keeping the baby in that position immediately after delivery/during her stay in the hospital (for facility deliveries)
Dry cord care	0-2	Nothing applied to the cord immediately after delivery or later until the cord fell off
Exclusive Breastfeeding in the past 24 hours	0-2	Baby not given anything other than breast milk, (except medicine or ORS) in the 24 hours prior to the interview
Have immunization card	9-11	Has a MCP card or immunization card for the baby
Polio (OPV3 or IPV) from card	9-11	(Recorded from card)
DPT3 from card	9-11	(Recorded from card)
Age-appropriate initiation of complementary feeding	9-11	First giving cereal-based semisolid food at age 6 – 8 months
Age appropriate frequency of complementary feeding	9-11	Baby given cereal-based semisolid food 3 – 4 times per day
Hand-washing at time of feeds	9-11	Mother washed her hands before feeding the baby the last time she fed him/he
Modern method of contraception used	9-11	Currently using one of the following methods:<br>1. Male sterilization<br>2. Female sterilization (TL)<br>3. IUD/Loop<br>4. Injectables<br>5. Condom/Nirodh<br>6. Contraceptive pills<br>7. Others (specify)

Supplementary Table 3: list of assets asked in each round

Electricity	Computer
Mattress	Refrigerator
Pressure Cooker	Watch or clock
Chair	Bicycle
Cot or bed	Motorcycle or scooter
Table	Animal-drawn cart
Electric fan	Car
Radio or transistor	Jeep
Black and white television	Water pump
Colour television	Thresher
Sewing machine	Tractor
Mobile phone	Gas stove
Any other type of phone	

Supplementary Table 4: Distribution of mothers of children aged 0-2 months by sociodemographic characteristics in each round of Community-based Household Surveys in Bihar, India, 2012-2017 in the 8 focus districts and 30 non-focus districts.

Variable	Round 2	Round 3	Round 4	Round 5	Round 6		Round 7		Round 8		Round 9	
	Focal	Focal	Focal	Focal	Focal	Other	Focal	Other	Focal	Other	Focal	Other
**Religion - Caste, %**												
Hindu, non-SC/ST*	1583 (60.1)	1580 (60.8)	1621 (61.1)	1587 (59.6)	2192 (62.5)	6882 (54.7)	2157 (61.8)	7075 (56.7)	2133 (61.3)	7066 (56.3)	2142 (61.4)	7081 (56.5)
Hindu, SC/ST	705 (26.4)	721 (27.0)	621 (24.4)	696 (27.1)	792 (23.6)	3399 (27.5)	813 (23.2)	3274 (26.3)	877 (25.7)	3375 (27.3)	876 (25.4)	3423 (27.6)
Non-hindu	315 (13.5)	302 (12.2)	357 (14.5)	320 (13.2)	457 (14.0)	1965 (17.8)	474 (14.9)	1856 (16.9)	431 (13.0)	1785 (16.4)	423 (13.1)	1737 (15.9)
Missing	-	-	-	-	-	-	-	-	-	-	-	-
**Literate, %**	862 (33.3)	944 (36.5)	983 (37.8)	933 (35.4)	1296 (37.9)	4850 (39.8)	1539 (44.9)	5479 (44.7)	1542 (45.1)	5764 (47.3)	1617 (46.7)	5880 (48.3)
**Gender of Focal Child. %**												
Female	1237 (47.0)	1241 (46.7)	1218 (46.7)	1264 (48.6)	1669 (48.5)	5878 (47.9)	1652 (47.8)	5879 (48)	1652 (47.9)	5802 (47.4)	1618 (47.0)	5889 (48)
Male	1366 (53.0)	1362 (53.3)	1381 (53.3)	1339 (51.4)	1772 (51.5)	6368 (52.1)	1792 (52.2)	6326 (52)	1789 (52.1)	6424 (52.6)	1823 (53.0)	6357 (52)
**Nuclear Family, %**	890 (33.9)	832 (31.3)	854 (31.9)	889 (33.7)	1446 (42.0)	4980 (41.3)	1156 (33.0)	4281 (35.9)	1289 (37.6)	4211 (34.9)	1239 (36.5)	4113 (34.6)
**House Type, %**												
Kachcha	1159 (46.0)	1160 (46.2)	1075 (42.8)	1090 (43.0)	1164 (33.6)	4666 (39.1)	885 (25.6)	4043 (34.5)	884 (26.2)	3707 (31.9)	758 (22.1)	3471 (30.4)
Pucca	411 (15.1)	442 (16.6)	458 (17.6)	436 (16.3)	699 (19.3)	2924 (22)	478 (13.2)	1907 (14.4)	423 (12.0)	1662 (12.8)	467 (12.9)	1815 (14)
Semi-Pucca	1033 (38.9)	1001 (37.2)	1066 (39.7)	1077 (40.7)	1578 (47.1)	4656 (38.9)	2081 (61.1)	6255 (51.2)	2134 (61.8)	6857 (55.3)	2216 (65.0)	6960 (55.6)
**Husband's Education, %**												
No Education	1175 (44.6)	1068 (40.3)	1067 (40.8)	1113 (42.2)	1484 (42.9)	5254 (42.9)	1338 (38.5)	4681 (39.2)	1408 (40.5)	4730 (39)	1339 (38.2)	4920 (40)
Primary Education (0-8 years)	756 (29.6)	793 (31.5)	770 (30.4)	797 (32.0)	960 (29.1)	2860 (24.6)	990 (29.3)	3033 (25)	843 (25.4)	2828 (23.7)	814 (24.8)	2421 (20.5)
Secondary Education (9-12 years)	533 (20.3)	582 (22.1)	598 (22.3)	544 (20.1)	755 (20.6)	3194 (25.1)	793 (22.6)	3322 (26.3)	808 (23.0)	3223 (25.5)	838 (23.4)	3234 (25.6)
Higher Education (>12 years)	106 (4.3)	121 (4.6)	124 (4.8)	114 (4.1)	196 (5.8)	812 (6.3)	225 (6.7)	896 (7.1)	206 (5.9)	876 (7)	227 (6.4)	1025 (8.3)
Missing	33 (1.4)	39 (1.5)	40 (1.6)	35 (1.5)	46 (1.6)	126 (1.1)	98 (3.0)	273 (2.4)	176 (5.2)	569 (4.7)	223 (7.1)	646 (5.6)
**Women's Education, %**												
No Education	1741 (66.7)	1659 (63.5)	1616 (62.2)	1670 (64.6)	2152 (62.3)	7436 (60.5)	1927 (55.7)	6840 (56.3)	1917 (55.5)	6584 (54)	1832 (53.6)	6438 (52.4)
Primary Education (0-8 years)	523 (20.2)	521 (20.5)	553 (21.4)	561 (21.7)	668 (20.1)	2167 (18.5)	728 (22.0)	2369 (19.6)	753 (22.6)	2499 (20.8)	696 (20.5)	2283 (19.1)
Secondary Education (9-12 years)	298 (11.5)	356 (13.5)	360 (13.6)	308 (11.3)	511 (14.5)	2218 (17.6)	637 (18.1)	2478 (20.2)	645 (18.4)	2625 (21.2)	766 (21.9)	2910 (23.6)
Higher Education (>12 years)	41 (1.6)	64 (2.5)	70 (2.7)	64 (2.4)	110 (3.2)	425 (3.4)	152 (4.2)	518 (4)	126 (3.5)	518 (4)	147 (4.1)	615 (4.9)
Missing		3 (0.1)				0 (0)		0 (0)		0 (0)		0 (0)
**Number of Kids, %**												
1	720 (27.6)	779 (30.1)	696 (26.8)	671 (26.0)	869 (24.5)	3268 (26.7)	986 (28.5)	3407 (27.9)	908 (26.2)	3237 (26.7)	934 (26.8)	3373 (27.8)
2	733 (28.4)	693 (26.3)	707 (27.4)	684 (26.6)	882 (25.3)	3256 (26.7)	912 (26.4)	3332 (27.2)	926 (27.2)	3415 (28.1)	958 (27.7)	3365 (27.7)
3	541 (20.6)	501 (19.1)	518 (19.6)	589 (22.0)	704 (20.8)	2469 (20.2)	718 (21.0)	2422 (19.9)	724 (21.3)	2611 (21.4)	703 (20.7)	2645 (21.4)
4+	609 (23.3)	630 (24.5)	678 (26.2)	659 (25.4)	986 (29.3)	3253 (26.4)	828 (24.1)	3044 (24.9)	883 (25.4)	2963 (23.8)	846 (24.8)	2863 (23.1)
**Women's Age, years, median(IQR)**	24.1 (21.1 - 27.3)	24.0 (21.0 - 26.8)	24.0 (21.2 - 27.2)	24.1 (21.2 - 27.1)	24.0 (21.1 - 27.0)	24.0 (21.2 - 26.6)	23.0 (20.1 - 25.5)	23.3 (20.6 - 26.0)	22.8 (20.0 - 25.5)	23.0 (20.3 - 25.6)	22.4 (19.9 - 25.2)	22.7 (20.1 - 25.2)
**Number of Assets, median(IQR)**	4.4 (2.76 - 6.46)	4.35.0 (2.73.1 -	4.35.2 (2.73.3 - 6.47.4)	4.35.1 (2.73.4 -	4.55.4 (2.93.6 - 6.57.5)	4.45.2 (2.83.4 -	5.66.5 (4.70 - 7.68.8)	5.76.5 (3.84.5 – 8.09.1)	5.76.7 (4.81 - 7.89.2)	6.17.0 (4.5.0 - 8.39.6)	6.37.5 (4.65.5 -	6.88.0 (4.85.7 -
**Household Size, median(IQR)**	6.7 (4.9 - 9.5)	6.8 (5.0 - 9.2)	6.8 (5.1 - 9.2)	6.7 (4.9 - 9.2)	6.4 (4.6 - 8.7)	6.2 (4.5 - 8.6)	6.2 (4.5 - 8.6)	6.5 (4.7 – 8.9)	6.6 (4.8 - 9.0)	6.6 (4. - 8.9)	6.4 (4.6 - 8.8)	6.5 (4.7 – 8.9)
**Number of Kids, median(IQR)**	1.8 (1.0 - 2.9)	1.8 (1.0 - 3.0)	1.8 (1.0 - 3.1)	1.9 (1.0 - 3.0)	2.0 (1.0 - 3.3)	1.9 (1.0 - 3.1)	1.9 (1.0 - 3.2)	1.8 (1.0 - 3.0)	1.8 (1.0 - 2.9)	1.8 (1.0–2.9)	1.9 (1.0 - 3.0)	1.8 (1.0 - 2.9)

*SC: scheduled caste, ST: scheduled tribe

Supplementary Table 5: Demographics of mothers of children aged 9-11 months by round of Community-based Household Surveys in Bihar, India, 2012-2017 in the 8 focus districts and 30 non-focus districts.

Variable	Round 2 Focal	Round 3 Focal	Round 4 Focal	Round 5 Focal	Round 6 Focal	Round 6 Other	Round 7 Focal	Round 7 Other	Round 8 Focal	Round 8 Other	Round 9 Focal	Round 9 Other
**Religion - Caste, %**												
Hindu, non-SC/ST*	1587 (60.1)	1520 (58.4)	1613 (61.2)	1533 (57.7)	2209 (62.9)	6964 (55.7)	2211 (63.2)	7139 (57)	2145 (61.7)	7199 (57.4)	2179 (62.7)	7167 (57.5)
Hindu, SC/ST	705 (27.0)	762 (28.6)	673 (25.9)	717 (27.7)	748 (22.4)	3387 (27)	790 (23.0)	3242 (26.5)	866 (25.1)	3156 (25.4)	858 (25.2)	3324 (26.5)
Non-hindu	311 (12.9)	321 (13.1)	313 (12.9)	353 (14.6)	483 (14.6)	1896 (17.3)	443 (13.9)	1824 (16.6)	430 (13.2)	1870 (17.2)	404 (12.2)	1749 (16)
Missing	-	-	-	-	-	-	-	-	-	-	-	6 (0)
**Literate, %**	815 (31.1)	818 (31.7)	858 (33.1)	982 (37.6)	1286 (37.5)	4608 (38.4)	1447 (41.8)	5290 (43.2)	1559 (45.8)	6025 (49.4)	1528 (44.6)	5785 (47)
**Gender of Focal Child, %**												
Female	1270 (49.0)	1272 (48.9)	1228 (47.0)	1243 (47.4)	1607 (46.9)	5748 (47)	1636 (47.4)	5761 (47.4)	1645 (47.7)	5901 (47.8)	1643 (47.9)	5794 (46.9)
Male	1333 (51.0)	1331 (51.1)	1371 (53.0)	1360 (52.6)	1833 (53.1)	6499 (53)	1808 (52.6)	6444 (52.6)	1796 (52.3)	6324 (52.2)	1798 (52.1)	6452 (53.1)
**Nuclear Family, %**	1025 (38.9)	1017 (38.9)	1008 (38.4)	1015 (38.9)	1593 (45.5)	5740 (47.3)	1428 (41.2)	5128 (43.1)	1454 (42.1)	4739 (39.7)	1423 (41.6)	4896 (41.3)
**House Type, %**												
Kachcha	1087 (43.3)	1150 (46.0)	1124 (44.7)	1020 (40.4)	1210 (34.9)	4657 (39.2)	849 (24.8)	4005 (34.4)	864 (24.8)	3598 (31.1)	743 (22.1)	3288 (28.5)
Pucca	392 (14.4)	432 (16.4)	447 (16.9)	483 (17.7)	689 (18.6)	2978 (22.6)	549 (15.0)	1943 (14.6)	448 (12.7)	1808 (14.1)	464 (12.9)	1991 (15.8)
Semi-Pucca	1124 (42.3)	1021 (37.6)	1028 (38.4)	1100 (41.9)	1541 (46.5)	4612 (38.3)	2046 (60.3)	6257 (51)	2129 (62.5)	6819 (54.8)	2234 (65.0)	6967 (55.6)
**Husband's Education, %**												
No Education	1164 (45.2)	1208 (45.8)	1113 (42.5)	1092 (41.5)	1483 (42.4)	5314 (43)	1324 (38.2)	4672 (39.3)	1350 (38.7)	4462 (36.8)	1382 (39.7)	4812 (39.6)
Primary Education (0-8 years)	738 (28.8)	671 (27.0)	784 (30.7)	746 (29.8)	981 (29.7)	2861 (24.6)	1002 (30.3)	3088 (25.7)	857 (25.7)	2873 (24.2)	806 (24.0)	2446 (20.8)
Secondary Education (9-12 years)	541 (20.2)	562 (20.9)	562 (21.2)	602 (22.5)	761 (21.3)	3171 (25.2)	823 (22.7)	3334 (25.8)	853 (24.0)	3355 (26.5)	779 (22.0)	3299 (26)
Higher Education (>12 years)	106 (3.6)	118 (4.5)	105 (4.0)	135 (5.0)	169 (5.0)	768 (6.1)	198 (5.6)	835 (6.8)	208 (6.2)	971 (7.8)	255 (7.6)	1006 (7.9)
Missing	54 (2.2)	44 (1.8)	35 (1.6)	28 (1.2)	46 (1.6)	133 (1.1)	97 (3.1)	276 (2.5)	173 (5.3)	564 (4.7)	219 (6.7)	683 (5.7)
**Women's Education, %**												
No Education	1788 (68.9)	1787 (68.4)	1741 (66.9)	1621 (62.4)	2161 (62.7)	7675 (61.9)	2017 (58.8)	7028 (57.7)	1901 (54.8)	6313 (51.7)	1927 (55.8)	6542 (53.9)
Primary Education (0-8 years)	489 (19.0)	447 (17.6)	483 (18.7)	560 (21.6)	679 (20.4)	2228 (19.2)	704 (20.7)	2311 (19.2)	674 (20.0)	2567 (21.3)	683 (20.8)	2210 (18.6)
Secondary Education (9-12 years)	279 (10.2)	322 (12.2)	327 (12.5)	341 (12.9)	516 (14.5)	1959 (15.8)	586 (16.6)	2359 (19)	712 (20.6)	2765 (22.4)	679 (19.2)	2844 (22.4)
Higher Education (>12 years)	47 (1.8)	47 (1.8)	48 (1.9)	81 (3.1)	84 (2.4)	385 (3.1)	137 (3.8)	507 (4)	154 (4.6)	580 (4.6)	152 (4.2)	650 (5.1)
**Number of Kids, %**												
1	724 (28.0)	697 (26.6)	702 (26.7)	772 (29.7)	923 (26.4)	3278 (27)	912 (26.0)	3403 (27.9)	1003 (28.9)	3675 (30.1)	966 (27.8)	3418 (28.1)
2	694 (26.7)	693 (26.7)	753 (29.0)	673 (26.3)	902 (25.9)	3317 (27.2)	906 (26.6)	3361 (27.5)	898 (26.0)	3332 (27.3)	951 (28.0)	3438 (28.2)
3	520 (20.0)	529 (20.2)	525 (20.4)	551 (20.9)	687 (19.7)	2417 (19.6)	711 (20.7)	2506 (20.6)	718 (21.1)	2445 (20)	705 (20.5)	2616 (21.1)
4+	665 (25.3)	684 (26.5)	619 (23.9)	607 (23.1)	928 (27.9)	3235 (26.2)	915 (26.7)	2935 (24.1)	822 (23.9)	2773 (22.6)	819 (23.7)	2774 (22.6)
**Women's Age, years, median(IQR)**	24.6 (21.6 - 27.9)	24.6 (21.8 - 27.8)	24.3 (21.5 - 27.5)	24.3 (21.5 - 27.4)	24.2 (21.6 - 27.1)	24.3 (21.6-27.1)	23.9 (21.1 - 26.6)	23.9 (21.2-26.6)	23.2 (20.6 - 25.8)	23.3 (20.6-25.9)	23.1 (20.3 - 25.6)	23.3 (20.6-25.9)
**Number of Assets, median(IQR)**	4.10 (2.4 - 6.43)	4.82 (2.53.0 - 6.92)	4.25.0 (2.63.3 - 6.27.1)	4.4 5.2 (2.83.5 – 7.56.6)	4.55.4 (2.83.5 - 67.6)	4.45.1 (2.73.3- 6.77.6)	56.4 (3.84.5 - 7.78.9)	5.66.4 (3.74.3-	56.8 (4.91 - 8.19.5)	67.2 (4.35.1- 8.59.8)	6.37.5 (4.55.4 - 8.49.9)	6.77.8 (4.75.6- 8.910.5)
**Household Size, median(IQR)**	6.3 (4.5 - 8.6)	6.3 (4.6 - 8.8)	6.3 (4.6 - 8.6)	6.3 (4.5 - 8.6)	5.9 (4.3 - 8.1)	5.8 (4.2-8.1)	6.1 (4.5 - 8.6)	5.9 (4.3-8.3)	6.0 (4.4 - 8.4)	6.1 (4.4-8.6)	5.9 (4.3 - 8.2)	5.9 (4.3-8.3)
**Number of Kids, median(IQR)**	1.8 (1.0 - 3.0)	1.9 (1.0 - 3.1)	1.8 (1.0 - 2.9)	1.8 (1.0 - 2.9)	1.9 (1.0 - 3.2)	1.8 (1-3.1)	1.9 (1.0 - 3.1)	1.8 (1-3)	1.8 (1.0 - 2.9)	1.7 (1-2.9)	1.8 (1.0 - 2.9)	1.8 (1-2.9)

*SC: scheduled caste, ST: scheduled tribe
